# Diagnostic value of ^18^F-FDG PET/MRI for staging in patients with endometrial cancer

**DOI:** 10.1186/s40644-020-00357-4

**Published:** 2020-10-22

**Authors:** Hideaki Tsuyoshi, Tetsuya Tsujikawa, Shizuka Yamada, Hidehiko Okazawa, Yoshio Yoshida

**Affiliations:** 1 Department of Obstetrics and Gynecology, University of Fukui, 23-3 Matsuoka-Shimoaizuki, Eiheiji-cho, Yoshida-gun, Fukui, 910-1193 Japan; 2 Biomedical Imaging Research Center, University of Fukui, Fukui, Japan

**Keywords:** ^18^F-FDG PET/MRI, Contrast-enhanced CT, Contrast-enhanced MRI, Preoperative staging, Endometrial cancer

## Abstract

**Background:**

Preoperative accurate assessment of endometrial cancer can assist in the planning of additional surgical options, and in predicting the prognosis. The aim of the present study was to evaluate the diagnostic potential of non-contrast PET/MRI with ^18^F-fluorodeoxyglucose (^18^F-FDG) for assessment in preoperative staging of endometrial cancer.

**Methods:**

Thirty-six patients with biopsy-proven endometrial cancer underwent preoperative ^18^F-FDG PET/MRI, contrast-enhanced CT (ceCT) and pelvic dynamic contrast-enhanced MRI (ceMRI) for initial staging. The diagnostic performance of ^18^F-FDG PET/MRI and ceMRI for assessing the extent of the primary tumor (T stage), and ^18^F-FDG PET/MRI and ceCT for assessing nodal (N stage) and distant (M stage) metastasis, was evaluated by two experienced readers. Histopathological and follow-up imaging results were used as the gold standard. The McNemar test was employed for statistical analysis.

**Results:**

Accuracy for T status was 77.8 and 75.0% for ^18^F-FDG PET/MRI and ceMRI, respectively. Patient-based accuracy for detecting regional nodal and distant metastasis was 91.3 and 81.8% for ^18^F-FDG PET/MRI, and 87.0 and 81.8% for ceCT. None of these parameters was statistically significant (*p* > 0.05). Lesion-based sensitivity, specificity and accuracy for detecting regional nodal metastasis were 100, 96.9 and 97.0% for ^18^F-FDG PET/MRI, and 14.3, 97.6 and 93.3% for ceCT; sensitivity was statistically significant (*p* < 0.05).

**Conclusions:**

Non-contrast ^18^F-FDG PET/MRI, which combines the individual advantages of PET and MRI, offers a high diagnostic value equivalent to that of ceMRI for assessment of the primary tumor, and equivalent to that of ceCT for the assessment of nodal and distant metastatic staging, in patients with endometrial cancer. These findings suggest that ^18^F-FDG PET/MRI might provide an alternative diagnostic strategy to conventional imaging modalities in the preoperative staging of endometrial cancer.

**Supplementary information:**

**Supplementary information** accompanies this paper at 10.1186/s40644-020-00357-4.

## Introduction

Endometrial cancer is the most common gynecological malignancy in developed countries, and its incidence continues to increase. Because endometrial cancer is staged postoperatively, the primary treatment is determined by predicting the FIGO classification [1]. Imaging examinations are thus indispensable in planning optimal treatment, for advanced disease as well as early-stage disease confined to the uterus; however, the FIGO classifications do not currently include imaging findings.

Standard treatment for early-stage disease is surgical resection, including hysterectomy and bilateral salpingo-oophorectomy. However, accurate assessment of the primary tumor, lymph nodes and distant metastasis can assist in the planning of additional surgical options such as pelvic or para-aortic lymphadenectomy or radical hysterectomy, and in predicting the prognosis [2].

In these evaluations of local disease, magnetic resonance imaging (MRI) offers better diagnostic performance than transvaginal ultrasonography (US) and could be considered the reference standard [3]. In particular, contrast-enhanced MRI (ceMRI) could provide additional diagnostic value particularly regarding myometrial invasion [4]. Positron emission tomography (PET), particularly with ^18^F-fluorodeoxyglucose (^18^F-FDG) as a tracer that reflects cellular metabolism, has been shown to be worth consideration alongside conventional imaging modalities. For the detection of lymph node and distant metastasis, ^18^F-FDG PET/computed tomography (CT) could be more useful than conventional imaging such as CT or MRI [5]; however, limited data are available for assessment of local disease [6].

The new integrated modality of ^18^F-FDG PET/MRI provides high soft-tissue contrast along with functional imaging of ^18^F-FDG uptake, without using gadolinium-based contrast agent, and has shown potentially better diagnostic performance than ^18^F-FDG PET/CT for gynecologic cancers [7–9]. In endometrial cancer, fusion of PET and MRI has revealed significantly higher accuracy for T staging compared with ^18^F-FDG PET/CT, and comparable accuracy for N staging compared with ^18^F-FDG PET/CT, suggesting that integrated PET/MRI has a possible diagnostic role to play, whereas integrated PET/MRI has not been well studied as yet [10].

Therefore, the aim of our study was to evaluate the diagnostic value of non-contrast ^18^F-FDG PET/MRI for whole-body tumor staging of patients with endometrial cancer, and to compare the diagnostic accuracy of ^18^F-FDG PET/MRI with that of ceCT and ceMRI.

## Material and methods

### Patients

We retrospectively reviewed the medical records of 53 patients with pathologically proven endometrial cancer who were treated at our institution between February 2016 and November 2018. Of these, 36 patients (mean age, 61.2 years; age range, 38–86 years) who had undergone ^18^F-FDG PET/MRI, ceCT, and pelvic dynamic ceMRI for initial staging based on the Japanese Imaging Guidelines of the Japan Radiological Society were included in the present study [11, 12]. Written informed consent was obtained from all patients prior to the imaging procedures. The characteristics of excluded patients are listed in Supplementary Table 1. All patients had completed ^18^F-FDG PET/MRI, ceCT and ceMRI within 3 months prior to treatment. The maximum interval among ^18^F-FDG PET/MRI, ceCT and ceMRI was 70 days (mean, 13.6 days; range, 1–70 days). Of the 36 patients, 33 underwent total abdominal or laparoscopic hysterectomy and 3 underwent radical hysterectomy; 31 patients underwent bilateral and 2 underwent unilateral salpingo-oophorectomy; 3 patients underwent bilateral salpingectomy; 23 patients underwent pelvic and 14 underwent para-aortic lymphadenectomy; 11 patients underwent partial omentectomy; and 1 patient underwent neoadjuvant chemotherapy. Patients were followed up for at least 1 year and 10 months after treatment based on the guidelines for treatment of uterine body neoplasm of the Japan Society of Gynecologic Oncology [12, 13]. Of the 36 patients, 6 had recurrent disease such as liver or lung metastasis, intra-pelvic masses, or carcinomatous peritonitis that was the cause of death in 2 of these 6 patients. Recurrent disease was suspected based on the subjective symptoms or elevated tumor markers, and diagnosed using CT, ^18^F-FDG PET/CT or PET/MRI.

### ^18^F-FDG PET/MRI

#### Whole-body PET/MRI

Patients fasted for at least 4 h prior to intravenous injection of 200 MBq of ^18^F-FDG. Fifty minutes after the injection, patients were transferred to a whole-body 3.0-T PET/MR scanner (Signa PET/MR, GE Healthcare, Waukesha, WI, USA). Anatomical coverage was from the vertex to the mid-thigh. PET acquisition was performed in 3D mode with 5.5 min/bed position (89 slices/bed) in 5–6 beds with a 24-slice overlap. A 2-point Dixon 3D volumetric interpolated T1-weighted fast spoiled gradient echo sequence was acquired at each table position and was used to generate MR attenuation correction (MR-AC) maps. Dixon-based MR-AC classifies body tissues into soft tissue, fat, and air. PET data were reconstructed with ordered subset expectation maximization (OSEM), selecting 14 subsets and 3 iterations, and post-smoothing with a 3-mm Gaussian filter. Reconstructed images were then converted to semiquantitative images corrected by the injected dose and the subject’s body weight (= standardized uptake value [SUV]).

#### Pelvic PET/MRI

After whole-body scanning and a brief break for urination, the patients were repositioned in the PET/MR scanner. The pelvic PET scan was performed as a 3D acquisition in list mode with 10 min/bed position (89 slices/bed) in 1–2 beds with a 24-slice overlap. The regional PET data were reconstructed with OSEM selecting 16 subsets and 4 iterations, and post-smoothing with a 4-mm Gaussian filter. The reconstructed images were then converted to SUV images. For pelvic MRI, T2-weighted images were acquired in the sagittal, transaxial and coronal planes, using the following T2-weighted image parameters: TR 4000–7000 ms, TE 90 ms, section thickness 4 mm, section overlap 0 mm, flip angle 100°, FOV 240 × 240 mm, matrix 384 × 384, two excitations, and bandwidth 83.3 kHz. High-resolution DW images were then obtained in the sagittal or transaxial plane with b-values of 0 and 800 s/mm^2^. A 2D RF excitation pulse and 180° refocusing pulse were used to reduce the FOV in the phase-encoding direction while simultaneously suppressing signal from fat. The imaging parameters were as follows: TR 4000 ms, TE 62.8 ms, section thickness 4 mm, section spacing 0 mm, flip angle 90°, FOV 240 × 120 mm (phase FOV = 0.5), matrix 96 × 128, 8 excitations, and bandwidth 250 kHz.

#### Dynamic Contrast-Enhanced (DCE) MRI

Pelvic MRI was performed using a 3-T clinical scanner (Discovery MR750, GE Healthcare, Waukesha, WI) in 27 patients. To delineate the anatomy of the pelvis prior to pelvic DCE-MRI, T2-weighted imaging was performed in the sagittal, transaxial, and coronal planes. The following T2-weighted image parameters were used: TR 3200–6000 ms, TE 60–85 ms, section thickness 4 mm, interval 1 mm, flip angle 111°, FOV 240 × 240 mm, matrix 320 × 224, two excitations, echo train length 10, and bandwidth 62.5 kHz. For DCE-MRI, a sagittal 3D fast spoiled-gradient-recalled T1-weighted sequence using the Dixon method with fat suppression (LAVA Flex, GE Healthcare) was used with the following parameters: TR 5.0 ms, TE 1.3 ms, section thickness 3 mm, flip angle 12°, FOV 260 × 260 mm, matrix 320 × 192, 1 excitation, and bandwidth 166.7 kHz. After non-contrast images were acquired, 0.1 mmol/kg of gadolinium-diethylenetriamine pentaacetic acid were injected at a rate of 2 ml/s using a contrast injector, followed by a 20-ml saline flush. Image sets were acquired at multiple phases, at 45, 80 and 120 s after initiation of the injection. In nine patients, DCE-MRI was performed at other institutes using 1.5-T clinical scanners (Magnetom Aera, Siemens Healthineers, or Signa HDe, GE Healthcare).

### Contrast-Enhanced (CE) CT

CT examinations covering the chest, abdomen and pelvis were performed using a 64-slice multidetector CT scanner (Discovery CT 750HD; GE Medical Systems, Milwaukee, WI) before and after intravenous administration of nonionic iodinated contrast material (iopamidol, Iopamiron 300; Schering, Berlin, Germany).

### Image interpretation

Images were analyzed on a dedicated workstation (Advantage Workstation 4.6, GE). Two board certificated radiologists/nuclear medicine physicians, each with double certifications and specialized in gynecological imaging, evaluated the ^18^F-FDG PET/MRI, ceCT and ceMRI images retrospectively and in consensus. The images were evaluated for the following: (a) presence of the primary tumor; (b) tumor extension into the myometrium, cervical stroma, uterine serosa or adnexa, vagina or parametrium, urinary bladder or rectum mucosa; (c) pelvic or para-aortic lymph nodes; (d) distant metastasis. Both readers were blinded to the results of other imaging studies, histopathologic findings and clinical data. Each dataset was reviewed with the consensus of the two readers after a minimum interval of 3 weeks to avoid any decision threshold bias due to reading-order effects. For MRI interpretation, several previous standard criteria related to primary tumor and nodal or distant metastatic staging of endometrial cancer were used as the reference criteria [3]. Swollen lymph nodes larger than 1 cm in short-axis diameter were graded as malignant. For ^18^F-FDG PET/MRI interpretations, the classification of lymph nodes as cancer-positive was based on the presence of focally appreciable metabolic activity above that of normal muscle; or asymmetric metabolic activity greater than that of normal-appearing lymph nodes at the same level in the contralateral pelvis, in a location that corresponded to the lymph node chains on the CT or MRI images, with reference to a previous report [7, 8]. Furthermore, the presence of a central unenhanced area suggesting central necrosis or peripheral low attenuation suggesting a fatty hilum within lymph nodes was considered a benign sign. Tumor invasion of neighboring structures was decided primarily on the basis of the CT or MRI findings, with reference to the ^18^F-FDG PET findings.

### Reference standard

Histopathological correlation regarding locoregional extension of the primary tumor was available in all 36 patients, and was used as the standard of reference for T staging. For nodal staging, the histopathological results (*n* = 22) were used as the gold standard. Because clinical standards of patient management did not require surgery or sampling of the detected lesions, we used a modified reference standard for patients without histopathological sampling, which took into account all prior and follow-up imaging. A decrease in size under treatment (*n* = 1, interval: 2 months) was regarded to indicate malignancy. For M staging, the histopathological results were used (*n* = 11).

### Statistical analysis

The McNemar test was used to determine the statistical significance of differences in the accuracy of T, N and M staging as determined by PET/MRI, ceCT and ceMRI. Statistical analysis was performed with PRISM software (Versions 6.0; GraphPad, San Diego, CA). Differences at *p* < 0.05 were considered to be statistically significant.

## Results

### Patient characteristics

According to the revised International Federation of Gynecology and Obstetrics (FIGO) criteria [1], the T stage was classified as atypical endometrial hyperplasia in 2 patients, pT1a in 17, pT1b in 7, pT2 in 4, pT3a in 3 and pT3b in 3. The histopathologic types of the primary tumors were atypical endometrial hyperplasia (*n* = 2), endometrioid adenocarcinoma (Grade 1 (*n* = 18), Grade 2 (*n* = 5), and Grade 3 (*n* = 3)), serous carcinoma (*n* = 6) and carcinosarcoma (*n* = 2). The N stage was classified as N0 in 21 patients, N1 in 2 and N2 in 1. The M stage was classified as M0 in 8 patients and M1 in 3 including the omentum and inguinal lymph nodes. The demographic data for the 36 patients are listed in Table 1.

**Table 1:** Patient characteristics

Patient	Age	Histology	Pathological stage	PET/MRI stage	ceMRI & ceCT stage
1	75	serous	T3bN2M0	T3bN2M0	T3bN1M0
2	53	G3	T1bN0M0	T1bN0M0	T1bN0M0
3	68	G1	T1aNXM0	T1aNXM0	T1aNXM0
4	68	serous	T3aNXM1	T3aNXM1	T3aNXM1
5	51	G3	T2N1M0	T2N1M0	T2N0M0
6	70	serous	T1aN0M0	T1aN0M0	T1aN0M0
7	69	G1	T1aN0M0	T1bN0M0	T1bN0M0
8	38	AH	AH	T1bNXM0	T1bNXM0
9	66	CS	T1aN0M0	T1aN0M0	T1aN0M0
10	47	G1	T3aNXM0	T3aNXM0	T3aNXM1
11	58	G2	T1aN0M0	T1aN0M0	T1aN0M0
12	67	G1	T1bN0M0	T1bN0M0	T1bN0M0
13	68	serous	T3bN0M1	T1aN0M0	T1bN0M0
14	85	G2	T1bNXM0	T1aNXM0	T1aNXM0
15	41	G1	T1aNXM0	T1aNXM0	T1aNXM0
16	61	serous	T1bN0M0	T1bN0M0	T1bN0M0
17	65	G1	T2N0M0	T2N0M0	T2N0M0
18	42	AH	AH	AH	AH
19	49	G1	T2N0M0	T2N0M0	T2N0M0
20	42	G1	T1aN0M0	T1aN0M0	T1aN0M0
21	50	G2	T2N0M0	T1aN0M0	T1aN0M0
22	83	serous	T3bNXM1	T2NXM0	T1bNXM1
23	58	G1	T1aNXMX1	T1aNXMX1	T1aNXMX
24	75	G1	T1aN0M0	T1aN0M0	T1aN0M0
25	76	G1	T1aN0M0	T1aN0M0	T1aN0M0
26	54	G1	T1aN0M0	T1aN0M0	T1aN0M0
27	62	G1	T1aN0M0	T1aN0M0	T2N0M0
28	59	G1	T1aN0M0	T1aN0M0	T1aN0M0
29	50	G1	T1aNXM0	T1aNXM0	T1aNXM0
30	58	G1	T1aN0M0	T1bN0M0	T2N0M0
31	86	G3	T1bN0M0	T1bN1M0	T1bN0M0
32	51	G1	T1aNXM0	T1aNXM0	T1aNXM0
33	74	CS	T3aNXM0	T2NXM0	T2NXM0
34	68	G2	T1bN0M0	T1bN0M0	T1bN0M0
35	63	G2	T1bN0M0	T1bN1M0	T1bN1M0
36	54	G1	T1aNXM0	T1aNXM0	T1aNXM0

Underline indicates over- or under-diagnosis

*G* grade, *AH* atypical endometrial hyperplasia, *CS* carcinosarcoma

### Primary tumor detection

PET/MRI and ceMRI detected 97.2% (35/36) of the primary tumors (no significant difference, *p* = 1).

### T staging

**Fig. 1:**
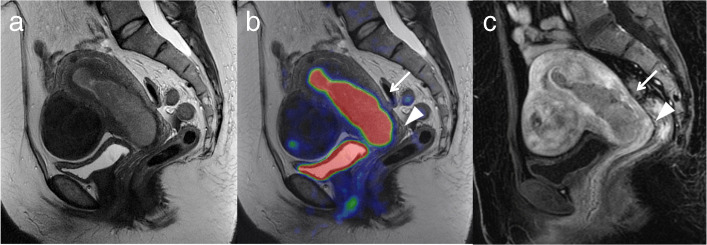
A 51-year-old woman with endometrial cancer invading 50% or more of the myometrium and cervical stroma (pT2). **a**. Sagittal T2-weighted MRI shows a large mass occupying the uterine cavity. **b**. Sagittal T2-weighted PET/MRI shows invasion of 50% or more of the myometrium (arrow) and cervical stroma (arrowhead). **c**. Sagittal T1-weighted dynamic contrast-enhanced MRI in the early phase shows the invasion of 50% or more of the myometrium without sub-endometrial enhancement (arrow) and cervical stroma (arrowhead). Histopathological examination of the surgical specimen was consistent with the imaging findings

The overall accuracy of T staging for PET/MRI and ceMRI was 77.8% (28/36) and 75.0% (27/36), respectively (Table 2; no significant difference, *p* = 1). PET/MRI overstaged the actual T stage in three patients (8.3%) and understaged it in five (13.9%), whereas ceMRI overstaged it in four patients (11.1%) and understaged it in five (13.9%). PET/MRI incorrectly classified three T1a tumors as T1b, whereas ceMRI incorrectly classified two T1a tumors as T1b and two T1a tumors as T2. Moreover, PET/MRI incorrectly classified one T1b tumor as T1a, one T2 tumor as T1a, one T3a tumor as T2 and two T3b tumors as T1a and T1b, whereas ceMRI incorrectly classified one T1b tumor as T1a, one T2 tumor as T1a, one T3a tumor as T2 and two T3b tumors as T1b and T2. The sensitivity, specificity and accuracy for detecting invasion of the myometrium were 92.9, 86.4 and 88.9% for PET/MRI, and 92.9, 81.8 and 86.1% for ceMRI, respectively (no significant difference, *p* = 1). The sensitivity, specificity and accuracy for cervical stroma were 85.7, 100 and 97.2% for PET/MRI and 71.4, 93.1 and 88.9% for ceMRI, respectively (no significant difference, *p* = 0.248). The accuracy for detecting invasion of the uterine serosa, adnexa, vagina and parametrium were 100, 91.7, 97.2 and 97.2%, respectively, for each of PET/MRI and ceMRI. No invasion of the urinary bladder or rectum mucosa was detected. Figure 1 shows representative images for T staging.

**Table 2:** Comparison of PET/MRI with ceMRI and ceCT for patient-based T, N and M staging

	PET/MRI	ceMRI and ceCT
Primary tumor		
Sensitivity	97.2% (35/36)	97.2% (35/36)
T staging		
Accuracy	77.8% (28/36)	75.0% (27/36)
≥50% myometrial invasion		
Sensitivity	92.9% (13/14)	92.9% (13/14)
Specificity	86.4% (19/22)	81.8% (18/22)
Accuracy	88.9% (32/36)	86.1% (31/36)
Invasion of cervical stroma		
Sensitivity	85.7% (6/7)	71.4% (5/7)
Specificity	100% (29/29)	93.1% (27/29)
Accuracy	97.2% (35/36)	88.9% (32/36)
Invasion of uterine serosa		
Sensitivity	100% (1/1)	100% (1/1)
Specificity	100% (35/35)	100% (35/35)
Accuracy	100% (36/36)	100% (36/36)
Invasion of adnexa		
Sensitivity	25.0% (1/4)	25.0% (1/4)
Specificity	100% (32/32)	100% (32/32)
Accuracy	91.7% (33/36)	91.7% (33/36)
Invasion of vagina		
Sensitivity	50.0% (1/2)	50.0% (1/2)
Specificity	100% (34/34)	100% (34/34)
Accuracy	97.2% (35/36)	97.2% (35/36)
Invasion of parametria		
Sensitivity	0% (0/1)	0% (0/1)
Specificity	100% (35/35)	100% (35/35)
Accuracy	97.2% (35/36)	97.2% (35/36)
N staging		
Accuracy	91.3% (21/23)	87.0% (20/23)
Metastatic pelvic lymph node		
Sensitivity	100% (2/2)	50% (1/2)
Specificity	90.5% (19/21)	95.2% (20/21)
Accuracy	91.3% (21/23)	91.3% (21/23)
Metastatic para-aortic lymph node		
Sensitivity	100% (1/1)	0% (0/1)
Specificity	100% (13/13)	100% (13/13)
Accuracy	100% (14/14)	92.9% (13/14)
M staging		
Sensitivity	33.3% (1/3)	66.7% (2/3)
Specificity	100% (8/8)	87.5% (7/8)
Accuracy	81.8% (9/11)	81.8% (9/11)

### N staging

The overall accuracy of N staging for PET/MRI and ceCT was 91.3% (21/23) and 87.0% (20/23), respectively (no significant difference, *p* = 1; Table 2). PET/MRI overstaged the actual N stage in two patients (8.7%), whereas ceCT overstaged it in one patient (4.3%), and understaged it in two (8.7%). PET/MRI incorrectly classified two N0 lymph nodes as N1, and ceCT incorrectly classified one N0 lymph node as N1, one N1 lymph node as N0 and one N2 lymph node as N1. Patient-based sensitivity, specificity and accuracy for detecting pelvic lymph node metastasis were 100, 90.5 and 91.3% for PET/MRI, and 50.0, 95.2 and 91.3% for ceCT, respectively (no significant difference, *p* = 0.480). Patient-based accuracy for detecting para-aortic lymph node metastasis was 100% for PET/MRI and 92.9% for ceMRI (no significant difference, *p* = 1). Lesion-based sensitivity, specificity and accuracy for detecting pelvic and para-aortic nodal metastasis were 100, 96.9 and 97.0% for ^18^F-FDG PET/MRI, and 14.3, 97.6 and 93.3% for ceCT, respectively. Sensitivity was statistically significant (*p* = 0.041 < 0.05), but specificity and accuracy were not significant (*p* = 1 and *p* = 0.182, respectively) (Table 3).

**Table 3:** Comparison of PET/MRI and ceCT for lesion-based nodal metastasis

	PET/MRI	ceCT
Sensitivity	100% (7/7)	14.3% (1/7)
Specificity	96.9% (123/127)	97.6% (124/127)
Accuracy	97.0% (130/134)	93.3% (125/134)

### M staging

**Fig. 2:**
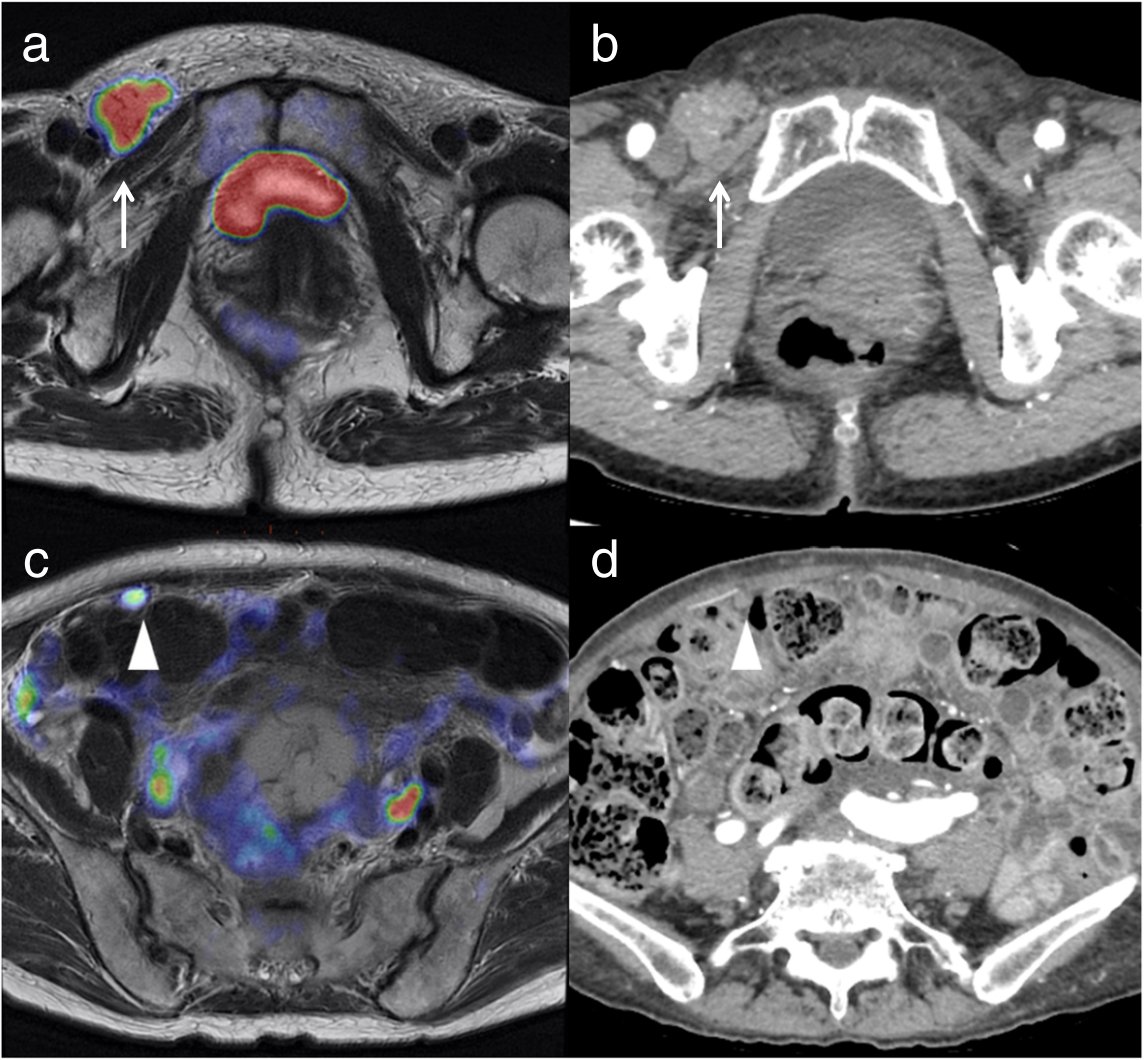
A 68-year-old woman with endometrial cancer and metastasis to an inguinal lymph node and the omentum (M1). **a**. Axial T2-weighted PET/MRI shows swelling of the right inguinal lymph node and high FDG uptake (arrow). **b**. The diameter of the node is > 10 mm (arrow) on contrast-enhanced CT. **c**. Axial T2-weighted PET/MRI shows omental dissemination with high FDG uptake (arrowhead) **d**. The diameter of the omental dissemination is > 10 mm (arrowhead) on contrast-enhanced CT. Histopathologic examination confirmed cancer involvement in the lymph node and omental nodule

The sensitivity, specificity and accuracy of M staging were 33.3, 100 and 81.8% for PET/MRI, and 66.7, 87.5 and 81.8% for ceCT, respectively (no significant difference, *p* = 0.480; Table 2). PET/MRI understaged the actual M stage in two patients (18.2%), whereas ceCT overstaged it in one patient (9.1%) and understaged it in another (9.1%). PET/MRI incorrectly classified two M1 tumor nodes in the omentum as M0, whereas ceCT incorrectly classified one M1 tumor in the omentum as M0 and one M0 tumor in the omentum as M1. Both PET/MRI and ceCE could detect inguinal lymph node metastasis. Figure 2 shows representative images for M staging, including metastasis to an inguinal lymph node and the omentum.

## Discussion

To the best of our knowledge, this is the first study to investigate the diagnostic value of non-contrast ^18^F-FDG PET/MRI, which has the diagnostic performance of both PET and MRI, for the staging of endometrial cancer in comparison with conventional imaging modalities such as ceMRI and ceCT. For T, N and M staging, the accuracy of ^18^F-FDG PET/MRI was equivalent to those of ceMRI and ceCT. This finding suggests that ^18^F-FDG PET/MRI might provide an alternative diagnostic strategy to conventional imaging modalities in the preoperative staging of endometrial cancer.

To optimize decision-making for additional surgical options (such as pelvic or para-aortic lymphadenectomy or radical hysterectomy) in the treatment in endometrial cancer it is important to identify the extent of disease prior to surgery. MRI is useful for evaluating the extent of local disease and its overall staging accuracy has been reported to be 85–95%. In evaluating myometrial invasion, the addition of dynamic ceMRI to T2WI has been reported to improve accuracy, and is considered the reference standard for preoperative detection of deep myometrial invasion [4]. However, contrast agents may induce anaphylactic shock or nephrogenic systemic fibrosis and cannot be used for patients with impaired renal function or allergy, which suggests that an alternative diagnostic strategy is needed for these patients. ^18^F-FDG PET/CT was comparable to ceMRI for predicting myometrial invasion, and was superior to that modality for identifying cervical invasion in a study of 318 patients with endometrial cancer [6]. In terms of PET/MRI, Kitajima et al. reported that fusion of PET and MRI had significantly higher accuracy for T staging compared with ^18^F-FDG PET/CT [10]. In the present study, the accuracy for identifying myometrial and cervical invasion was 88.9 and 97.2% for integrated ^18^F-FDG PET/MRI, and 86.1 and 88.9% for ceMRI. None of these parameters was statistically significant (*p* > 0.05), which suggests that integrated ^18^F-FDG PET/MRI might be an alternative modality to ceMRI.

In terms of the assessment of adnexae, a study of 58 patients with endometrial cancer and ovarian malignancy found that preoperative pelvic MRI had sensitivity of 51.7%, which is far below its specificity of 99.9% [14]. In that study, 43.1% of the lesions were occult carcinomas that could barely be identified by preoperative imaging or surgical exploration. ^18^F-FDG PET/CT offered low diagnostic value in differentiating ovarian malignancies due to low ^18^F-FDG uptake, leading to false-negative results [15]. Nevertheless, the usefulness of ^18^F-FDG PET/MRI has been reported: fused ^18^F-FDG PET/MRI showed higher sensitivity (94%, and specificity of 100%) for the characterization of ovarian tumors compared with those of MRI and ^18^F-FDG PET/CT [16]. In terms of the assessment of vaginal and parametrial invasion, there have been no reports in endometrial cancer. In cervical cancer, MRI provides rather low sensitivity for the assessment of vaginal and parametrial invasion, but high specificity compared with CT and clinical assessment. Also for cervical cancer, ^18^F-FDG PET/CT has been reported to have lower accuracy (53.3%) [17], whereas ^18^F-FDG PET/MRI correctly identified the T stage (85%) [18]. In the present study, both ^18^F-FDG PET/MRI and ceMRI showed low sensitivity and high specificity for detecting the adnexal, vaginal and parametrial invasion due to the presence of micro-invasion and these results were consistent with those of previous studies. None of these parameters was statistically significant (*p* > 0.05), which suggests that^18^F-FDG PET/MRI may also be applied to endometrial cancer with ovarian malignancies, or vaginal and parametrium invasion, in place of ceMRI.

The FIGO classification system divides lymph node metastasis into pelvic lymph node metastases and para-aortic lymph node metastases. In a systematic review of the Cochrane database, lymphadenectomy did not decrease the risk of death or recurrence, and appeared to increase the risk of surgical-related complications in women with low risk of recurrence; however, in patients at intermediate or high risk of recurrence, combined pelvic and para-aortic lymphadenectomy may improve overall survival [19]. Accurate preoperative detection of lymph node metastasis as well as local disease may thus be necessary to determine the optimal treatment and to improve prognosis. A previous study found that for the detection of lymph node metastasis, ^18^F-FDG PET/CT had a slightly low sensitivity of ~ 70% and high specificity of > 90%, and may be superior to CT or MRI [5]. In the assessment of N staging in endometrial cancer using fused ^18^F-FDG PET/MRI, Kitajima et al. reported that patient-based sensitivity, specificity and accuracy for detecting pelvic nodal metastasis were 100, 96.3 and 96.7% for both fused ^18^F-FDG PET/MRI and ^18^F-FDG PET/ceCT, and 66.7, 100 and 96.7% for MRI, respectively [10]. In addition, Shih et al. reported that SUVmax using integrated ^18^F-FDG PET/MRI was significantly higher in tumors with lymph node metastasis [20]. In terms of M staging, for the detection of distant metastasis such as to supraclavicular or mediastinal lymph nodes or bone (IVB), a previous study reported sensitivity and specificity of more than 90% for ^18^F-FDG PET/CT, which indicates its value compared with conventional imaging [21]. These results suggest that ^18^F-FDG PET/MRI can be superior to ceCT and comparable to ^18^F-FDG PET/CT in the assessment of N and M staging. However, in the present study, there were no significant differences in N and M staging between ^18^F-FDG PET/MRI and ceCT, although previous reports have suggested the superiority of ^18^F-FDG PET/MRI compared with the conventional modalities in these situations of other cancers [18, 22]. The possible reason for the lack of difference could partly be due to the small number of events and samples in our study. Further studies with a larger sample size are needed.

On the basis of these previous studies, ^18^F-FDG PET/MRI has a higher diagnostic value than ^18^F-FDG PET/CT and is comparable to ceMRI for the assessment of T staging, whereas ^18^F-FDG PET/MRI has a higher diagnostic value than ceCT and is comparable to ^18^F-FDG PET/CT for the assessment of N or M staging. In the present study, ^18^F-FDG PET/MRI showed comparable diagnostic value to ceMRI and ceCT for the staging of endometrial cancer: the accuracy of T, N and M staging by^18^F-FDG PET/MRI was 77.8, 91.3 and 81.8%; and the accuracy of T staging by ceMRI and that of N and M staging by ceCT was 75.0, 87.9 and 81.8%, respectively. The diagnostic accuracy of our results is slightly lower than in previous studies because there were high rates of micro-invasion or micro-metastasis in the present patients, and the total sample number was small. Taking these facts into consideration, our study demonstrates the potential diagnostic value of non-contrast ^18^F-FDG PET/MRI for the staging of endometrial cancer.

This study had several limitations. First, it was a retrospective study, and not all MRI examinations were performed at our institution. However, our readers re-evaluated the images from other hospitals and were blinded to the initial imaging findings. Second, the sample size of our study was relatively small, and further prospective studies are needed. Third, we could not evaluate the histopathological correlation with imaging in 13 (36.1%) patients who had not undergone lymphadenectomy. Thus, we performed node-specific comparison between imaging and histopathology in all other patients.

## Conclusion

For the assessment of primary, nodal and metastatic staging in patients with endometrial cancer, integrated ^18^F-FDG PET/MRI without a gadolinium-based contrast agent shows potential diagnostic value compared with the conventional modalities of ceCT, ceMRI or ^18^F-FDG PET/CT. ^18^F-FDG PET/MRI might provide an alternative diagnostic strategy to conventional imaging modalities in the preoperative staging of endometrial cancer, particularly for patients with severe renal dysfunction or allergy to contrast agents.

## Supplementary information

**Additional file 1: Supplementary Table 1.** Characteristics of patients excluded from the present study

## Data Availability

The datasets used and/or analyzed during the current study are available from the corresponding author on reasonable request.

## Abbreviations

ceMRI: Contrast-enhanced magnetic resonance imaging
ceCT: Contrast-enhanced computed tomography
US: Ultrasonography
^18^F-FDG PET: ^18^F-fluorodeoxyglucose Positron emission tomography
SUV: Standardized uptake value
